# Common and proper nouns in mild Alzheimer's disease

**DOI:** 10.1002/alz70858_096688

**Published:** 2025-12-24

**Authors:** Sonia Zehtab, Leila Ghasisin

**Affiliations:** ^1^ Esfahan University of Medical Sciences, Esfahan, Esfahan, Iran (Islamic Republic of)

## Abstract

**Background:**

Disturbance in naming accuracy and reaction time (RT) is one of the early symptoms of Alzheimer's disease. Naming performance can be considered a diagnostic key in the early stages of Alzheimer's disease (AD), which has remained diagnostically challenging. Although most of the studies in this field have been conducted on the naming accuracy of common nouns, others have shown that proper nouns are more sensitive for detecting the onset of AD. This study aims to compare the naming of common and proper nouns.

**Method:**

Eighty pictures of common and proper nouns (40 items each) were presented to 18 healthy older adults and 18 people with mild Alzheimer's disease using DMDX software on a laptop computer. The patients’ responses were transcribed into a pre‐designed form, and their reaction times were captured by DMDX.

**Result:**

Study results indicated a significant difference in the number of errors and RTs between proper and common nouns in patients with mild Alzheimer's disease (*p*‐value=), implying that proper nouns may be more sensitive to mild AD. Moreover, patients with mild Alzheimer's had more problems in common and proper nouns than healthy older adults.

**Conclusion:**

This study demonstrated that individuals with mild AD experienced greater difficulty recalling proper nouns, which were found to be more susceptible to the effects of AD.

